# Giant conjunctival melanoma in a paranoid schizophrenic man: A case report

**DOI:** 10.1016/j.amsu.2021.01.069

**Published:** 2021-01-26

**Authors:** Tommy Supit, Pujisriyani, Subiyakto, Trilaksana Nugroho, Alifiati Fitrikasari, Najatullah

**Affiliations:** a Department of General Surgery, Faculty of Medicine Diponegoro University, Dr. Kariadi General Hospital, Jl. Dr. Sutomo No. 16, Randusari, Semarang Selatan, Semarang, Jawa Tengah, 50244, Indonesia; b Department of Plastic Surgery, Faculty of Medicine Diponegoro University, Dr. Kariadi General Hospital, Jl. Dr. Sutomo No. 16, Randusari, Semarang Selatan, Semarang, Jawa Tengah, 50244, Indonesia; c Department of Oncologic Surgery, Faculty of Medicine Diponegoro University, Dr. Kariadi General Hospital, Jl. Dr. Sutomo No. 16, Randusari, Semarang Selatan, Semarang, Jawa Tengah, 50244, Indonesia; d Department of Ophthalmology, Faculty of Medicine Diponegoro University, Dr. Kariadi General Hospital, Jl. Dr. Sutomo No. 16, Randusari, Semarang Selatan, Semarang, Jawa Tengah, 50244, Indonesia; e Department of Psychiatry, Faculty of Medicine Diponegoro University, Dr. Kariadi General Hospital, Jl. Dr. Sutomo No. 16, Randusari, Semarang Selatan, Semarang, Jawa Tengah, 50244, Indonesia

**Keywords:** Cancer, Orbital, Conjunctival, Melanoma, Paranoid, Schizophrenia

## Abstract

**Introduction:**

and importance: Conjunctival melanoma (CM) is a rare and potentially lethal ocular tumor. As with any oncologic disease, early diagnosis and appropriate treatment of CM is paramount to limit morbidity and increase life expectancy. However, patients with severe mental disability with social isolation are usually presented in late-stage disease.

**Case presentation:**

This report presents a case of a 55-year-old man with paranoid schizophrenic man with an extraordinarily large CM due to neglect. The patient suffered from complete left eye blindness with no clinical and radiological evidence of metastasis.

**Clinical dicussion:**

Clinicians must bear in mind the limited patient compliance and family support of mentally-ill patients that restricts treatment modalities that would have otherwise been applicable for cooperative patients. The importance multidisciplinary approach, choosing the simpler but effective surgical technique should be prioritized.

**Intervention and outcome:**

Left exenteration and tumor wide excision was performed. The left orbital defect was reconstructed using forehead flap and split-thickness skin graft (STSG). The uncooperative nature of the patient posed early post-operative challenges that necessitates subsequent operation to drain seroma. The patient was discharged 16-days after operation with acceptable cosmetic and clinical results. However, the patient failed to return to the clinic for longer post-operative evaluation.

**Conclusion:**

A multidisciplinary approach is mandatory to treat complex cases such as this report. Surgeons are advised to adopt simpler surgical approach that will require minimal self-care and should encourage family members to continuously support the patient.

## Highlights

• A case report of an extremely large conjunctival melanoma due to self-neglect caused by mental-illness.
• An interdisciplinary approach and correct selection of surgical technique is paramount for patients with minimal self-care and family support.
• Discussion of the inherent challenges in the perioperative management of oncoplastic patients with paranoid schizophrenia.

## 1 Introduction

Conjunctival melanoma (CM) is an uncommon tumor comprising 2% of all ocular tumors and 0.25% of all melanomas [1]. The risk factors for CM are not yet established however, the incidence of CM is increasing along with cutaneous melanoma, which suggests a possible association with ultraviolet light exposure [2]. The molecular pathogenesis of CM is more similar to cutaneous melanoma compared to its uveal counterpart. A study using exome sequencing detected mutations in BRAF, NRAS, NF1, EGFR, ALK, TERT, and APC oncogenes in CM [3]. Patients with CM usually complain of a painless brownish lesion on the surface of the eye, and occasionally ocular irritation or pain [4]. Differential diagnosis of CM include primary acquired melanosis, conjunctival nevi, local extension of uveal melanoma or melanocytoma, and distant metastasis of cutaneous melanoma [5]. This care report is reported in line with the surgical case report (SCARE) guideline [6].

Primary treatment of CM involves wide surgical excision followed by adjuvant therapy (i.e. cryotherapy, topical alcohol application, brachytherapy). However, systemic metastasis occurs in 19% of the patient within 3.4 years with no effective treatment for metastatic disease [5]. Disease recurrence, involvement of non-bulbar conjunctiva, medial bulbar conjunctiva, caruncle and plica semilunaris, tumor thickness of more than 2 mm, *de novo* origin, and nodular growth pattern are all risks for metastasis and mortality [5]. We present a neglected case of CM in a paranoid schizophrenic allowing it to grow into a very large size. We report a case of CM presented in a late-stage in a paranoid schizophrenic man with minimal self-care and family support. The importance of multidisciplinary approach, choosing the practical surgical approach, and the challenges in treating mentally ill patient are discussed.

## 2 Case report

**Fig. 1:**
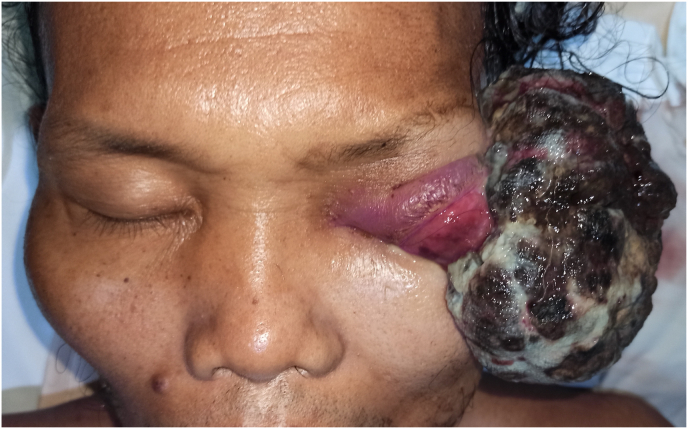
A large irregularly shaped tumor protruding from the left eye socket with outstretched extraocular muscles. The phthisic left globe cannot be visualized from outside.

A 55-year-old male presented in the outpatient clinic with a large and foul-smelling tumor growing out of his left eye since a year ago. The mass was initially a small black nodule on the temporal side of the limbus. Other than complete blindness of the left eye, there were no symptoms related to central nervous, cardiorespiratory, or gastrointestinal system. History ocular trauma, excessive sun exposure, and family with malignancy was denied. Physical examination revealed a baseball-sized irregular mass with dark brown to greyish discoloration protruding out of the left eye socket. The fungating tumor surface was partially covered with necrotic tissue, slough, and blood clot. Computed tomography (CT) scan with contrast demonstrated a mixed density mass with antero-posterior diameter of 7.7 cm and latero-lateral diameter of 7.8 cm, phthisic left globe, extra cavitary stretching of optic nerve, ophthalmic vein, and extraocular muscles (Fig. 1). There was no evidence of distant metastasis from head neck CT-scan, and pulmonary plain radiography.

The patient was reported to display an increasingly abnormal behavior within the last two years with reports of disorganized speech, self-talk, and difficulty in communication. The patient mostly lived alone or by the care of his siblings after his spouse and children left him. There was no history of medical treatment for the tumor or mental illness. The dominant symptoms at the time of admission and during hospital stay were auditory hallucinations, suspicious behavior, and delusion of persecution. The psychiatric assessment confirmed the diagnosis of paranoid schizophrenia (International Classification of Mental and Behavioral Disorders [ICD]-10 Code F20.0). He was prescribed Olanzapine 10 mg daily, Amitriptyline 100 mg daily, Trihexyphenidyl 1 mg daily, and Diazepam 15 mg daily along with routine psychotherapy throughout the hospital stay.

**Fig. 2:**
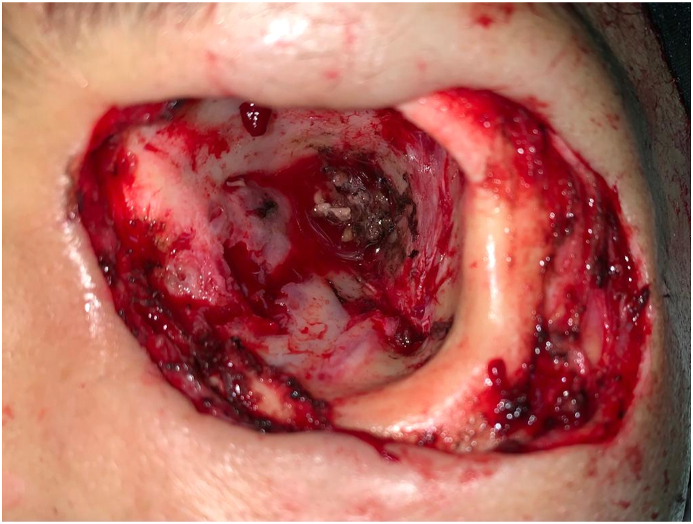
Left orbital defect post exenteration and wide excision revealing an empty eye socket with no bone defect.

The patient underwent left exenteration and wide excision that include the eye globe, eyelids, retrobulbar soft tissues, and periosteum with tumor-free margin confirmed by intraoperative frozen sections (Fig. 2). The left orbital defect was reconstructed using forehead flap and split-thickness skin graft (STSG) harvested from the left lateral thigh to cover the forehead wound (Fig. 3A). The surgery was performed board-certified specialists: one ophthalmologist, one oncologic surgeon, and two plastic surgeons. Pathologic analysis confirmed the diagnosis of invasive conjunctival melanoma (ICD-Oncology code 8720/3) with evidence of tumor invasion to the retrobulbar vasculature and fat tissue. Frozen sections revealed clear resection margins thus, no nonexcisional adjuvant therapy was applied. To facilitate postoperative drainage, a single 16 French tube was inserted at the posterolateral side of the left orbit.

**Fig. 3A:**
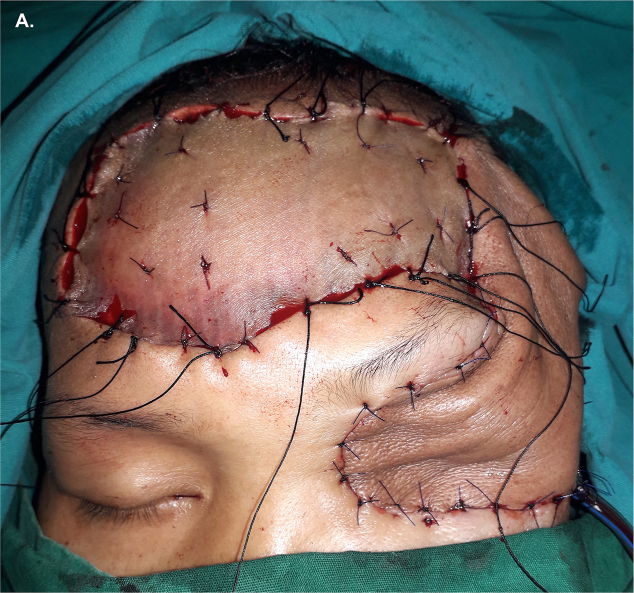
Post-reconstructive picture showing forehead flap and forehead defect closure with STSG.

However, 2 days later it was forcefully pulled out by the patient caused by his paranoid nature. Bilateral arm restraints were applied and additional intravenous Diazepam 10 mg was administered as necessary. Serohemorrhagic fluid was observed to be oozing from the previous drain tract saturating the gauze, requiring daily change. Bulging of the distal flap covering the orbit was noted one-week post-operation necessitating seroma drainage. The drainage was performed twice at day 10 and day 15 before the patient was finally discharged 16 days post-operation under stable psychiatric condition. Post-operation at day 21 showed vital forehead flap and STSG with satisfactory cosmetic results (Fig. 3B). Regrettably, this is the latest information we had regarding his postoperative condition since the patient only managed to came once for postoperative control 7 days after discharge (21 days post-operation).

**Fig. 3B:**
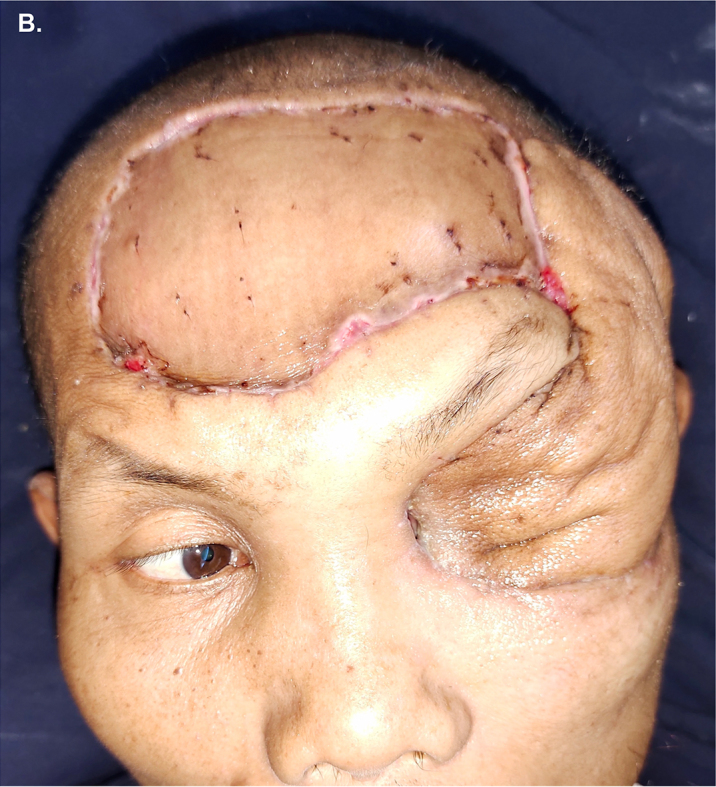
Appearance of the reconstructed defect 21-days post operation showing vital STSG and forehead flap. The excess tissue at the base of the flap was left intact to ensure flap vitality. Complete secondary healing of the STSG wound edges and cosmetical improvement of the flap is expected.

## 3 Discussion

A meta-analysis suggests the standardized incidence rate of melanoma in a patient with schizophrenia was significantly lower (0.71) compared to the healthy population [7]. Excess dopamine, enhanced natural killer cells, increased apoptosis rate, and modulation by antipsychotic drugs were some of the proposed hypotheses for this finding [8]. There is a trend for psychiatric patients with cancer to present later and with more advanced disease than the general population [9]. They seek medical help when symptoms become more pronounced in the later stages of the disease.

It is extremely rare for CM to be left to grow into very large because of its obvious early clinical signs. To our knowledge, this is the first case report CM with such magnitude. The necrotic tissue surrounding the tumor can be a result of cancer cell ischemia due to the rapid expansion of the tumor unsupported by angiogenesis. While the presence of noisome and air-bubble density within the tumor suggests an ongoing infection process. The patient presented with poor performance status with neglected self-care and with no supportive family members, and the condition is expected to resume throughout the duration of treatment and recovery.

This should alarm the clinicians to opt for the treatment modality that is safe and perhaps simpler for the patient to personally take care of. As in our case, efforts in contacting the patient and his family members had failed as of the creation of this manuscript. This predicament highlights the inherent challenge of treating oncoplastic patients with mental illness with limited family support.

The mainstay treatment of CM is wide local excision with “no-touch” technique followed with cryotherapy. Exenteration is justified for a large tumor with orbital and complete conjunctival invasion [4]. The postoperative defect, in this case, is what Kesting et al. described as type I defects where the resection involves the whole orbital content and periosteum without bony resection [10]. They suggested orbital coverage with STSG that can be followed by abutment placement and application of oculoplastic prosthesis [11]. We preferred forehead flap and STSG to cover the donor defect because it is a relatively quick procedure with acceptable outcomes.

Furthermore, it requires less demanding postoperative care compared to free flap and more durable for future external beam radiotherapy. The seroma formation within the hollowed orbit would have been mitigated with the use of tube drainage. However, it is evident from this case that its use is less reliable in an uncooperative patient. Temporalis muscle flap to fill in the empty eye socket would have been an excellent technique for this problem. Radial forearm flap is another alternative however, it is less ideal for this case since it is our best interest to create a wound that requires simple care.

## 4 Conclusion

Oncologic patients with psychiatric illness are usually presented with a more advanced stage disease. The main challenge in treating this group of patient lies in their level of compliance and self-care. Comprehensive treatment will be very hard to achieve without a multidisciplinary approach and the aid of supportive family members. Surgeons are advised to adjust their clinical decision based on the patient's performance status and to opt for the surgical technique that will allow optimal wound care.

## Provenance and peer review

Not commissioned, externally peer-reviewed.

## Consent

Written informed consent obtained from the patient for the purpose of publishing this case report. Information within the paper has been sufficiently anonymized not to cause harm to the patient or his family. A copy of signed informed consent is available for review by the Editor-in-Chief of this journal on request.

## Sources of funding

Nothing to declare.

## Ethical approval

Ethical approval exempted by our institution.

## Author contribution

Tommy Supit and Najatullah conceptualized the paper, performed perioperative patient care, follow-up, and manuscript drafting. Pujisriyani, Subiyakto, Trilaksana Nugroho performed the operation, validation of clinical history, investigations, and operative findings. Alifiati Fitrikasari performed psychiatric assessment, perioperative care and follow-up. All authors were involved in manuscript writing and approval for submission.

## Research registration

N/a.

## Guarantor

Tommy Supit

Department of General Surgery, Faculty of Medicine Diponegoro University, Dr. Kariadi General Hospital, Semarang, Indonesia.

## Declaration of competing interest

None declared.

## References

[1] Chang A.E., Karnell L.H., Menck H.R. (1998). The National Cancer Data Base report on cutaneous and noncutaneous melanoma: a summary of 84,836 cases from the past decade. The American College of Surgeons Commission on Cancer and the American Cancer Society. Cancer.

[2] Triay E., Bergman L., Nilsson B., All-Ericsson C., Seregard S. (2009). Time trends in the incidence of conjunctival melanoma in Sweden. Br. J. Ophthalmol..

[3] Swaminathan S.S., Field M.G., Sant D., Wang G., Galor A., Dubovy S.R. (2017). Molecular characteristics of conjunctival melanoma using whole-exome sequencing. JAMA Ophthalmol.

[4] Shields C.L., Shields J.A., Gündüz K., Cater J., Mercado G.V., Gross N. (2000). Conjunctival melanoma: risk factors for recurrence, exenteration, metastasis, and death in 150 consecutive patients. Arch. Ophthalmol..

[5] Agha R.A., Franchi T., Sohrabi C., Mathew G., Kerwan A., Thoma A. (2018). The SCARE 2020 guideline: updating consensus surgical CAse REport (SCARE) guidelines. Int. J. Surg..

[6] Wong J.R., Nanji A.A., Galor A., Karp C.L. (2014). Management of conjunctival malignant melanoma: a review and update. Expet Rev. Ophthalmol..

[7] Catts V.S., Catts S.V., O’Toole B.I., Frost A.D. (2008). Cancer incidence in patients with schizophrenia and their first-degree relatives - a meta-analysis. Acta Psychiatr. Scand..

[8] Goldacre M.J., Kurina L.M., Wotton C.J., Yeates D., Seagroatt V. (2005). Schizophrenia and cancer: an epidemiological study. Br. J. Psychiatry.

[9] Kisely S., Lawrence D., Kelly G., Pais J., Crowe E., Murph M. (2011). The interaction between melanoma and psychiatric disorder. Melanoma in the Clinic - Diagnosis, Management and Complications of Malignancy.

[10] Kesting M.R., Koerdt S., Rommel N., Mücke T., Wolff K.D., Nobis C.P. (2017). Classification of orbital exenteration and reconstruction. J. Cranio-Maxillofacial Surg..

[11] Cameron M., Gilbert P.M., Mulhern M.G., Sneddon K.J. (2005). Synchronous reconstruction of the exenterated orbit with a pericranial flap, skin graft and osseointegrated implants. Orbit.

